# Therapeutic Targeting Strategies of Cancer Stem Cells in Gastrointestinal Malignancies

**DOI:** 10.3390/biomedicines7010017

**Published:** 2019-03-10

**Authors:** Mohamad B. Sonbol, Daniel H. Ahn, Tanios Bekaii-Saab

**Affiliations:** Mayo Clinic Cancer Center, Mayo Clinic, Phoenix, AZ 85054, USA; Sonbol.mohamad@mayo.edu (M.B.S.); ahn.daniel@mayo.edu (D.H.A.)

**Keywords:** cancer stem cell, BBI-608, napabucasin, STAT3, pancreatic cancer, gastric cancer, colorectal cancer, Wnt/β-catenin

## Abstract

Cancer stem cells (CSCs) are thought to be a distinct population of cells within a tumor mass that are capable of asymmetric division and known to have chemoresistant characteristics. The description and identification of CSC models in cancer growth and recurrence has inspired the design of novel treatment strategies to overcome treatment resistance by targeting both CSCs and non-CSC tumor cells. Several cellular signaling pathways have been described as playing a role in the induction and maintenance of stemness in CSCs, such as the Wnt/β-catenin, Notch, STAT3, and Hedgehog pathways. In this review, we aim to review some of the ongoing CSC therapeutic targeting strategies in gastrointestinal malignancies.

## 1. Introduction

Cancer stem cells (CSCs) are thought to be a small and distinct population within the tumor mass that are capable of asymmetric division and known to have chemoresistant characteristics [1]. These cells acquire their chemo- and radioresistant features through different pathways that involve apoptosis and DNA repair mechanisms. [2].

In addition, upon exposure to conventional cytotoxic therapies, CSCs are capable of converting non-CSCs to CSC-like cells, which persist after treatment and could potentially serve as a mechanism for relapse following exposure to cytotoxic therapy. The description and identification of CSC models in cancer growth and recurrence has inspired the design of novel treatment strategies to overcome resistance by targeting both CSCs and non-CSC tumor cells [2]. Herein, we will review previous and ongoing treatment strategies aimed at targeting CSCs in gastrointestinal malignancies, including gastric and gastroesophageal cancers, colorectal cancer, pancreatic cancer, and hepatocellular carcinoma.

## 2. CSC Signaling Pathways

**Figure 1:**
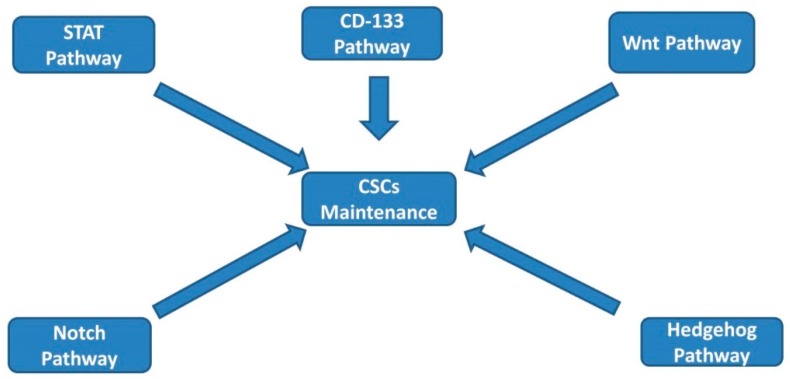
Selected signaling pathways involved in cancer stem cells maintenance. CSCs: cancer stem cells.

Several cellular signaling pathways have been described to play a role in the induction and maintenance of stemness in CSC. The signal transducers and activator of transcription 3 (STAT3) pathway plays an important role in regulating many physiological functions, such as inflammation, and proliferation in both normal and malignant tissue. Furthermore, the STAT3 pathway appears to be directly linked to CSCs, where its activation has been associated with the transformation of quiescent gastric CSCs into invasive gastric CSCs [3,4] Recently, interleukin (IL)-22, a cytokine that in humans is encoded by the *IL22* gene and highly expressed in pancreatic cancer cells, has been identified to promote pancreatic cancer stemness through STAT3 activation [5].The Notch pathway is one of the integral signaling pathways responsible for maintaining the undifferentiated state of CSCs [6,7]. Preclinical studies have demonstrated that deletion in genes that encode the Notch pathway ligand DLL1 (Delta-like ligand 1) or the DNA-binding protein RBJ-Jκ accelerated differentiation of pancreatic progenitor cells into endocrine cells [8]. Notch pathway components are highly expressed in pancreatic adenocarcinoma, suggesting that the Notch pathway may be involved in pancreatic CSCs’ self-renewal [9]. Furthermore, Notch signaling is thought to be associated with the acquisition of the epithelial-to-mesenchymal transition (EMT) phenotype and the generation of CSCs in pancreatic cancer [10]. EMT is an important process that causes epithelial cells to gain mesenchymal phenotypes, leading to high capacity for invasion and metastases of the cancer cells and associated with a poor clinical outcome [11]. The Wnt/β-catenin pathway has also been identified as a pathway that contributes to increased self-renewal and clonogenic potential of CSCs [12]. Studies have shown that abnormal Wnt signaling is related to early stage colon cancer as a result of mutations in genes encoding adenomatous polyposis coli (APC), β-catenin, or Axin [13]. Furthermore, Wnt signaling, through Wnt receptor Frizzled2 (Fzd2) and the ligand Wnt5a/b, are elevated in metastatic liver and colon cancer cell lines, and their expression was correlated with EMT [14]. Wnt activation and signaling is involved in the EMT process and contributes to tumor cell invasion and poor prognosis [2] (Figure 1). In addition to the abovementioned pathways, the Hedgehog signaling pathway is also believed to be related to the stemness state [15]. Normally, the Hedgehog pathway is linked to organ patterning, along with cell proliferation and differentiation processes. Furthermore, it is frequently activated in esophageal, gastric, and pancreatic cancers with upregulation of the Hedgehog ligands [16]. Additionally, it is found to be involved in both maintaining stemness and in the EMT process. Therefore, hedgehog inhibitors have been developed and investigated as potential therapeutic agents [17]. 

## 3. Napabucasin

Napabucasin (BBI-608, Boston Biomedical Inc., Cambridge, MA, USA) is an orally available first-in-class cancer stemness inhibitor that appears to target and inhibit gene transcription induced by STAT3 and cancer cell stemness properties [18]. Several clinical trials are investigating the safety and efficacy of BBI-608 across various gastrointestinal malignancies [6,19]. Below, we summarize results from the main clinical trials that have been completed or are ongoing (Table 1).

### 3.1. Pancreatic Cancer

Napabucasin was initially investigated a multi-center, single-arm, phase IB/II study in pancreatic adenocarcinoma (PDAC) [20]. In this study, 59 patients with metastatic pancreatic adenocarcinoma (mPDAC) were enrolled to receive napabucasin (240 mg twice daily) in combination with nab-paclitaxel and gemcitabine weekly, 3 out of 4 weeks until disease progression [20]. The most common observed adverse events (AEs) were fatigue and gastrointestinal related AEs, including diarrhea and nausea, which were acceptable and resolved upon treatment discontinuation. Among the 50 patients with evaluable disease, the disease control rate was 92%, with 2 complete responses (CR; 4%) and 26 partial responses (PR; 52%) [20]. Among all 59 patients enrolled, 1 year and 2 year overall survival (OS) rates were 46% and 13%, respectively. Given these promising results, this treatment combination is being further investigated in CanStem111P, an ongoing randomized international phase III trial where treatment-naïve patients with metastatic pancreatic adenocarcinoma are randomized to receive gemcitabine and nab-paclitaxel with or without napabucasin, with a primary endpoint of overall survival (NCT02993731).

### 3.2. Gastric and GEJ Cancers

In gastric and gastroesophageal junction (GEJ) cancer, napabucasin has demonstrated potential anti-tumor activity early in phase clinical trials. In a phase IB/II study of 46 patients with gastric/GEJ adenocarcinoma, the combination of napabucasin with paclitaxel showed a response rate of 31% (5/16) in taxane-naïve patients [21]. Interestingly, in patients that were previously treated with taxane-based chemotherapy, a promising response rate of 11% (2/19) was observed, suggesting a potential association of BBI-608 and taxane chemotherapy re-sensitization in taxane refractory disease [21]. These promising findings led to the BRIGHTER study, a randomized, double-blind, placebo-controlled phase III study that assessed the efficacy and safety of napabucasin in combination with paclitaxel in patients with treatment refractory advanced gastric and GEJ adenocarcinoma [19]. At the preplanned interim analysis at two thirds (380) of events, and based on the patient outcomes, the study was halted due to futility, with no safety signals. Median OS was 6.93 months vs. 7.36 months in the napabucasin vs. placebo arms (HR 1.01 (95% CI, 0.86–1.20), *p* = 0.8596), respectively. No significant difference in progression free survival (PFS) was observed, where the median PFS was 3.55 vs. 3.65 months in the napabucasin vs. placebo arms (HR 1.00 [95% CI, 0.84–1.17] *p* = 0.9679). Grade 3 or greater AEs were similar between the two treatment arms (69.2% in napabucasin vs. 59.7% in the placebo), with the exception of grade ≥3 diarrhea, which was 16.0% vs 1.4%, respectively [22]. Exploratory studies, including the subgroup analysis for phosphor-STAT3 elevation, are still pending. These results might provider further insight into selecting patients with gastric and GEJ adenocarcinoma who are likely to derive benefit from napabucasin.

### 3.3. Colorectal Cancers

In colorectal cancer (CRC), napabucasin has been investigated across several studies as a monotherapy and in combination with various chemotherapy regimens. BBI608-246 was a multi-arm phase I/II clinical trial that investigated napabucasin in combination with various standard therapeutic regimens for advanced gastrointestinal malignancies [23]. In a select cohort that included patients with metastatic CRC (mCRC), the primary objective was to determine the recommended phase II dose (RP2D) and evaluate the efficacy of napabucasin in combination with FOLFIRI (5-FU 400 mg/m^2^ intravenous bolus followed by infusional 5-FU 240,000 mg/m^2^ over 46 h), leucovorin 400 mg/m^2^ and irinotecan 180 mg/m^2^ every 14 days) +/− bevacizumab. Patients received napabucasin 240 mg twice daily (BID) with bi-weekly FOLFIRI +/− bevacizumab at 5 mg/kg until disease progression or other discontinuation criterion. Eighty two pretreated patients were included in the intention to treat analysis including 32 (39%) previously treated with FOLFIRI +/− bevacizumab [20]. Of the 82 patients, 48 received FOLFIRI and 34 FOLFIRI plus bevacizumab in combination with napabucasin. No dose-limiting or unexpected toxicity was observed. The most common AEs were gastrointestinal with grade 4 diarrhea in one patient, and 27 patients with grade 3 AEs including: diarrhea (15/27), fatigue (5), electrolyte imbalance (4), abdominal pain (1), vomiting (1), and weight loss (1). Disease control rate (DCR) was observed in 83 % (55 of 66) evaluable patients with 1 CR (1.5%), 13 PR (20%), and 41 SD (41%) [20]. CanStem303C (NCT02753127) is an ongoing randomized phase III clinical trial evaluating napabucasin in combination with FOLFIRI in adult patients with previously treated mCRC [24]. Napabucasin monotherapy has also been studied and reported on in a recently published phase III trial. The CO.23 trial evaluated the efficacy of napabucasin monotherapy vs. placebo in mCRC, which failed to demonstrate a significant difference in the napabucasin group survival (4.4 months (95% CI 3.7–4.9) vs. the placebo group (4·8 months (95% CI 4.0–5.3)) with an adjusted hazard ratio HR 1.13, 95% CI 0.88–1.46, *p* = 0.34. In a prespecified biomarker analysis, pSTAT3-positive patients experienced a significant survival benefit from napabucasin over placebo (median OS of 5.1 vs. 3 months, HR 0.41, *p* = 0.0025) [25].

### 3.4. Hepatocellular Carcinoma

The safety and efficacy of napabucasin in combination with standard of care therapy in patients with hepatocellular carcinoma (HCC) was reported in a phase Ib/II trial [23]. The study was an open-label trial in patients with advanced HCC who had not received prior systemic chemotherapy. The primary objective was to determine RP2D. In Arm 1, napabucasin was administered at 160 mg BID (dose level I) and at 240 mg BID (dose level II) in combination with sorafenib, and in arm 2, amcasertib (BBI-503, Boston Biomedical Inc.) was administered at 100 mg daily (dose level I) and at 200 mg QD (dose level II) in combination with sorafenib. The most common AEs were attributed to sorafenib included rash, grade 1/2 diarrhea, nausea, and vomiting. In Arm 1, the DCR was observed in all of the evaluable patients (6/6) (DCR was 67% in the ITT population). Median PFS was 24.9 weeks in the ITT population and 32.6 weeks in the evaluable patients [26].

## 4. Amcasertib

Amcasertib (BBI-503, Boston Biomedical Inc.) is another oral cancer stemness kinase inhibitor. It targets the serine–threonine stemness kinases, thereby inhibiting Nanog and other cancer stemness pathways [27]. In a phase 1 trial, amcasertib was given to 26 patients with advanced solid tumors (including 5 CRC, 2 HCC, 1 gastric/GEJ cancer, and 1 pancreatic neuroendocrine tumor), in a dose escalation fashion (10 to 450 mg once daily) [27]. The drug was well tolerated, with the main observed side effects being gastrointestinal-related. Grade 3 diarrhea was observed in 2 patients at 450 mg once daily. Of the 20 evaluable patients, 11 experienced stable disease. In the CRC expansion cohort, 47 patients with treatment refractory CRC were enrolled and received amcasertib monotherapy at 20 mg to 500 mg total daily. Most patients (*N* = 35, 75%) received amcasertib at RP2D, 300 mg once daily [28]. Similar to what was observed in the earlier study, the most common AEs were grade 1 to 2 diarrhea, abdominal pain, fatigue, and nausea/vomiting/anorexia. At the RP2D, grade 3 AEs were diarrhea (*N* = 3), fatigue (*N* = 3), nausea (*N* = 2), and weight loss (*N* = 1). Nanog expression (biomarker positive) was assessed in 39 (83%) patients. Based on Nanog expression, DCR was 56% in the biomarker positive patients compared to 13% in the biomarker negative patients (*p* = 0.040).

## 5. CART-133

In CSCs, CD133 is overexpressed and is thought to have a role in organizing cell membranes in normal tissues. It is expressed in approximately 50% of gastrointestinal malignancies, including HCC, PDAC, gastric cancer, and intrahepatic cholangiocarcinoma [29,30]. CD133 is also prognostic in HCC, where higher expression is associated with higher tumor stage and poor prognosis [31,32]. These features led to the development of CART-133, CD133-specific chimeric antigen receptor-modified T cells. CAR-T cell therapy has emerged as a tool for cancer treatment specifically in hematological malignancies [33]. Twenty three patients were enrolled (14 HCC, 7 PDAC, and 2 CRC) in a phase 1 study (NCT02541370) assessing safety and efficacy of CART-133 cell infusion in CD 133-positive tumors [34]. All enrolled patients had metastatic treatment refractory disease (treatment failure with two or more lines of therapy). The primary observed toxicities were anemia and thrombocytopenia, which were self-limited and resolved within one week. The acceptable cell dose was determined to be 0.5–2 × 106/kg, with the primary toxicity of a decrease in hemoglobin/platelet (≤grade 3) that is self-recovered within 1 week. Three (1 HCC and 2 non-HCC) of the 23 enrolled patients achieved partial response, with an additional 14 having stable disease. Median PFS was 5 months [34].

## 6. Other Agents in Development

Demcizumab (OncoMed Pharmaceuticals, OMP-21M18) is an anti-DLL4 (delta-like ligand 4) antibody that is being investigated for its inhibitory CSC activity, where it has been demonstrated to inhibit the Notch pathway. The drug was investigated in a double blind randomized 3 arm placebo-controlled phase 2 study (YOSEMITE trial) [35]. A total of 204 patients with metastatic PDAC were randomized to the combination of gemcitabine and nab-paclitaxel in addition to placebo (Arm 1), or two different doses of demcizumab (Arm 2: single 70-day truncated course of demciziumab; Arm 3: two 70-day truncated courses of demcizumab). The primary endpoint was PFS. The addition of demcizumab to the standard treatment did not improve PFS or OS in mPDAC [35]. 

The Hedgehog pathway has been believed to maintain the stemness state and to be involved in the EMT process. Therefore, Hedgehog inhibitors have been developed and investigated as a potential therapeutic agent in PDAC. However, dissimilar to the efficacy observed in other solid tumor malignancies [17], vismodegib failed to improve PFS or OS in combination with gemcitabine compared to the historical data for gemcitabine alone in a study of 23 patients with PDAC (2.8 and 5.3 months for mPFS and mOS, respectively) [36]. Confirmatory results were observed in a phase IB/II study of gemcitabine in combination with vismodegib, which did not show an improvement in PFS or OS [37]. Consistent with previous studies, saridegib (IPI-296), another Hedgehog inhibitor, failed to demonstrate a clinical benefit compared to placebo when added to gemcitabine in a phase 2 study of patients with untreated metastatic PDAC [37]. These results led to stopping the aforementioned trial and halting further development of Hedgehog inhibitors in metastatic PDAC.

## 7. Conclusions

CSCs are overexpressed across gastrointestinal malignancies and contribute to chemotherapy resistance, tumor invasion, and metastasis, and thus represent a potential target in the treatment in these diseases. Various novel agents aimed at inhibiting CSC pathways have shown promise in preclinical studies, but, contrary to these findings, they have not translated to meaningful clinical activity in patients. Tumor intrinsic and extrinsic characteristics, including the tumor stroma and its microenvironment, are barriers that are likely contributing to treatment resistance and tumor metastases. Ongoing clinical trials and their coinciding correlative studies will potentially allow us to better understand mechanisms of resistance and to identify and validate biomarkers that may allow us to better select patients likely to receive benefit from these class of agents.

## Figures and Tables

**Table 1:** Selected trials evaluating napabucasin in gastrointestinal malignancies ORR: Objective response rate; PR: Partial response; PD: Progressive disease; CR: Complete response; OS: overall survival; PFS: Progression-free survival.

Study ID	Phase	Treatment Administered	Number of patients	Outcome
**GEJ/Gastric**				
Shitara 2015 [1]	Phase 1	Napabucasin + paclitaxel	6	ORR 33.3% (2 PR)
Hitron 2014 [2]	Phase 1b dose escalation	Napabucasin + paclitaxel	24 patients with advanced cancers (5 with GEJ or gastric cancers)	ORR 40% (2/5 PR)
Becerra 2015 [3]	Phase Ib/II extension study	Napabucasin + paclitaxel	46 patients	ORR 15%; 11% (2/19) in patients with prior taxane; 31% (5/16) with no prior taxane exposure
Shah 2018 [4] (the BRIGHTER trial)	Phase III	Napabucasin + paclitaxel versus placebo	714 patients	Median OS 6.93 months vs. 7.36 in napabucasin vs. placebo (statistically not significant)
**Pancreatic**				
Bekaii-Saab 2018 [5]	Phase IB/II study	Napabucasin + nab-paclitaxel and gemcitabine	59 (50 evaluable patients)	2 CR (4%) and 26 PR (52%)
CanStem111P study (NCT02993731)	Phase III	Napabucasin + nab-paclitaxel and gemcitabine	Pending	primary endpoint of OS (results pending)
**CRC**				
Bendell 2017 [6]	Phase I/II	Napabucasin + FOLFIRI +/− bevacizumab	82 (66 evaluable patients)	1 CR (1.5%), 13 PR (20%), and 27 SD (41%)
CanStem303C (NCT02753127) [7]	Phase III	Napabucasin in combination with FOLFIRI	Pending	primary endpoint of OS (results pending)
**HCC**				
El-Rayes 2017 [8]	Phase I/II	Napabucasin + sorafenib	6	DCR 67%; PFS 24.9 weeks; OS 32.6 weeks
